# Ferroptosis-pyroptosis crosstalk signature as prognostic biomarkers and therapeutic targets in sepsis-induced ARDS

**DOI:** 10.3389/fcell.2026.1810674

**Published:** 2026-05-15

**Authors:** Lizhou Song, Haocheng Zhuang, Leibing Zhang, Hongda Bi

**Affiliations:** The First Affiliated Hospital of Naval Medical University, Shanghai, Yangpu, China

**Keywords:** ferroptosis-pyroptosis crosstalk, monocytes and macrophages, regulated cell death, sepsis-induced acute respiratory distress syndrome (ARDS), single-cell RNA sequencing (scRNA-seq)

## Abstract

**Background:**

Acute respiratory distress syndrome (ARDS) is a severe complication of sepsis with high mortality. Ferroptosis and pyroptosis are two distinct forms of regulated cell death that have been implicated in sepsis-induced organ dysfunction. However, the crosstalk between these 2 cell death pathways in sepsis-induced ARDS remains unclear.

**Methods:**

We analyzed gene expression data from sepsis and ARDS patients to identify ferroptosis-pyroptosis crosstalk genes. Single-cell RNA sequencing was performed to characterize cell type-specific expression patterns. Gene expression was validated using quantitative PCR and ELISA assays. Pathway enrichment analysis and protein-protein interaction networks were constructed to elucidate the molecular mechanisms.

**Results:**

We identified 10 ferroptosis-pyroptosis crosstalk genes with differential expression between ARDS and sepsis-only groups. Key genes including GPX4, GSDMD, SLC7A11, and CASP1 showed significant dysregulation in ARDS. Single-cell analysis indicated heterogeneous expression across immune cell populations, with enrichment of selected signals in myeloid cells. Pathway analysis indicated enrichment in inflammatory response, oxidative stress, and cell death pathways. PCR and ELISA validation confirmed the differential expression of these biomarkers.

**Conclusion:**

Ferroptosis-pyroptosis crosstalk genes serve as potential prognostic biomarkers and therapeutic targets in sepsis-induced ARDS. The identified gene signature provides insights into the pathophysiology and may guide the development of targeted interventions.

## Introduction

Sepsis is a life-threatening condition characterized by dysregulated host response to infection, leading to organ dysfunction and systemic inflammatory responses (Singer et al., 2016). It represents a major global health challenge, affecting millions of patients annually with substantial morbidity and mortality. ARDS represents one of the most severe complications of sepsis, with mortality rates exceeding 40% despite significant advances in critical care management (Force et al., 2012). The pathogenesis of sepsis-induced ARDS involves complex interactions among immune dysregulation, endothelial dysfunction, epithelial injury, and coagulation abnormalities (Thompson et al., 2017). Despite decades of research and advances in supportive care strategies including lung-protective ventilation and fluid management, the molecular mechanisms underlying sepsis-induced ARDS remain incompletely understood, and effective targeted therapies are lacking (Matthay et al., 2019).

Ferroptosis is an iron-dependent form of regulated cell death that is morphologically, biochemically, and genetically distinct from apoptosis, necroptosis, and other forms of cell death (Dixon et al., 2012). It is characterized by lethal accumulation of lipid peroxidation and reactive oxygen species (ROS) derived from iron-dependent enzymatic and non-enzymatic reactions (Stockwell et al., 2017). Key regulators of ferroptosis include glutathione peroxidase 4 (GPX4), which protects against lipid peroxidation by reducing lipid hydroperoxides to lipid alcohols, and the system xc-cystine-glutamate antiporter, particularly its catalytic subunit SLC7A11 (solute carrier family seven member 11) (Yang et al., 2014; Kagan et al., 2017). GPX4 utilizes glutathione as a cofactor, and cellular glutathione levels are maintained through cystine import *via* SLC7A11. Disruption of either GPX4 activity or cystine availability renders cells vulnerable to ferroptotic cell death (Friedmann Angeli et al., 2014). Ferroptosis has been implicated in various pathological conditions including ischemia-reperfusion injury, neurodegeneration, cancer, and inflammatory diseases (Doll et al., 2017; Gao et al., 2015).

Pyroptosis is a highly inflammatory form of programmed cell death mediated by the gasdermin protein family, particularly gasdermin D (GSDMD) (Shi et al., 2015; Liu et al., 2016). It is initiated by the activation of inflammatory caspases, primarily caspase-1 (CASP1) in the canonical inflammasome pathway and caspase-4/5/11 in the non-canonical pathway (Lamkanfi and Dixit, 2014; Kayagaki et al., 2015). Upon activation, these caspases cleave GSDMD to generate an N-terminal fragment (GSDMD-NT) that oligomerizes and forms pores in the plasma membrane, leading to cell swelling, membrane rupture, and release of pro-inflammatory cytokines including interleukin-1β (IL-1β) and IL-18 (Ding et al., 2016). The inflammasome complex, consisting of pattern recognition receptors such as NLRP3, the adaptor protein ASC, and pro-caspase-1, serves as the platform for caspase activation (Broz and Dixit, 2016). Pyroptosis plays crucial roles in host defense against pathogens but can also contribute to excessive inflammation and tissue damage in sepsis and other inflammatory diseases (Cheng et al., 2017; Kayagaki et al., 2011). The massive release of damage-associated molecular patterns (DAMPs) and inflammatory mediators during pyroptosis can amplify systemic inflammation and contribute to organ dysfunction (Krysko et al., 2011).

Recent evidence suggests complex crosstalk between ferroptosis and pyroptosis pathways in inflammatory conditions, with potential synergistic effects on cell death and tissue injury (Yu et al., 2020; Miao et al., 2022). Shared upstream regulators, common metabolic alterations, and reciprocal amplification mechanisms may link these 2 cell death modalities (Chen et al., 2021). For instance, lipid peroxidation products generated during ferroptosis can activate inflammasomes and trigger pyroptosis, while inflammatory mediators released during pyroptosis can inhibit antioxidant defenses and promote ferroptosis (Chu et al., 2016; Fang et al., 2020). Mitochondrial dysfunction, oxidative stress, and dysregulated iron metabolism represent potential convergence points for both pathways (Jiang et al., 2021; Wand et al., 2020). However, the specific role of ferroptosis-pyroptosis crosstalk in sepsis-induced ARDS has not been systematically investigated at the molecular, cellular, and systems biology levels. Understanding the molecular signatures of ferroptosis-pyroptosis interaction, their cell type-specific expression patterns, and their contribution to ARDS pathophysiology may reveal novel therapeutic targets and prognostic biomarkers (Stuart et al., 2019; Li et al., 2020). Such knowledge could facilitate the development of targeted interventions that simultaneously modulate both cell death pathways, potentially offering superior therapeutic efficacy compared to targeting either pathway alone (Xia et al., 2019).

## Methods

### Data collection and processing

Gene expression data from sepsis and ARDS patients were obtained from the Gene Expression Omnibus (GEO) database and ArrayExpress. Specifically, we analyzed the following datasets: (1) GSE65682 (bulk RNA-seq, n = 479 sepsis patients including 82 with ARDS, demographic details: mean age 58.3 ± 14.2 years, 56% male, primary infection sources documented); (2) GSE151263 (scRNA-seq from peripheral blood mononuclear cells, n = 6 sepsis patients [3 with ARDS, 3 sepsis-only], yielding 9,697 high-quality cells from ARDS patients and 11,139 cells from sepsis-only patients after quality control); (3) E-MTAB-7772 (additional bulk transcriptomic validation cohort, n = 156 samples). ARDS patients were defined per Berlin criteria based on available clinical annotations in each dataset. Sepsis-only controls were defined as patients meeting Sepsis-3 criteria without an ARDS diagnosis annotation in the respective database records. Single-cell RNA sequencing data were processed using Cell Ranger v7.0 for read alignment and gene quantification, followed by Seurat v4.3 for downstream analysis. Quality control criteria included: minimum 200 and maximum 6,000 detected genes per cell, mitochondrial gene percentage below 15%, and doublet removal using DoubletFinder. Normalization was performed using SCTransform to account for technical variation. Batch correction was applied using Harmony algorithm to integrate data from multiple sequencing runs and patient cohorts. Donor-level cell contributions are detailed in Supplementary Table S1, with sensitivity analyses using downsampled datasets (equalizing cell numbers through random sampling) performed to verify findings were not driven by unequal representation. Principal component analysis was performed, and the top 30 principal components were selected for downstream clustering based on elbow plot visualization.

### Ferroptosis-pyroptosis gene identification

Ferroptosis-related genes were systematically curated from the FerrDb database (version 2.0) and recent published literature through PubMed searches. A total of 382 ferroptosis-related genes were initially identified. Pyroptosis-related genes were obtained from Gene Ontology (GO:0070269) and pathway databases including KEGG and Reactome, yielding 156 pyroptosis-related genes. To identify crosstalk genes, we constructed protein-protein interaction networks using STRING database (v11.5) with a confidence score threshold of 0.7. Genes showing direct or indirect interactions between ferroptosis and pyroptosis modules were designated as crosstalk genes. We employed network topology analysis to calculate betweenness centrality, degree centrality, and closeness centrality for each gene. Genes with centrality scores in the top quartile and expression correlation coefficients exceeding 0.5 with genes in both pathways were selected as core crosstalk genes. This analysis was performed using Cytoscape v3.9 and the NetworkAnalyzer plugin. From the 10 identified crosstalk genes, we prioritized a four-gene panel (GPX4, GSDMD, CASP1, SLC7A11) for experimental validation based on three pre-specified quantitative criteria: (1) network centrality scores exceeding the 90th percentile across all crosstalk genes (betweenness centrality ≥0.15, degree centrality ≥12); (2) log2 fold-change magnitude greater than 1.5 in bulk RNA-seq data (GSE65682); and (3) detection in at least 30% of cells in single-cell analysis (GSE151263). Quantitative rankings for all 10 genes are provided in Supplementary Table S2.

### Single-cell RNA sequencing analysis

Single-cell data were analyzed using Seurat package (version 4.3) in R statistical environment (version 4.2.0). Cell clustering was performed using the shared nearest neighbor (SNN) algorithm with resolution parameter set to 0.8, generating 18 distinct cell clusters. Cell type annotation was performed using canonical markers: CD3D/CD3E/CD3G for T cells, CD19/M4A1 for B cells, CD14/FCGR3A for monocytes, CD68/CD163 for macrophages, and EPCAM for epithelial cells. Manual curation was complemented with automated annotation using SingleR against the Human Primary Cell Atlas reference. Differential gene expression analysis was conducted using pseudo-bulk aggregation to address pseudo-replication, where cells were aggregated by patient before statistical testing. We employed edgeR with robust dispersion estimation for pseudo-bulk differential expression, followed by mixed-effects models with patient as a random effect. Both patient-level and cell-level results are reported separately with clear distinctions in interpretation. Significance threshold was set at adjusted p-value <0.01 and log2 fold change >0.5. Pseudotime trajectory analysis was performed using Monocle 3 to investigate transcriptional continuum along an inferred developmental trajectory. Importantly, pseudotime represents a computational ordering of cells along a transcriptional gradient inferred from cross-sectional data and does not establish temporal causality or actual disease staging. UMAP (Uniform Manifold Approximation and Projection) dimensionality reduction was employed for visualization with n_neighbors = 30 and min_dist = 0.3 parameters. Cell-cell communication analysis was conducted using CellChat to identify ligand-receptor interactions between cell types.

### Pathway enrichment analysis

Gene Ontology (GO) and KEGG pathway enrichment analyses were performed using clusterProfiler package (version 4.6) in R. We analyzed biological processes, molecular functions, and cellular components from GO database. For KEGG analysis, we focused on pathways related to cell death, immune response, and oxidative stress. Multiple testing correction was applied using Benjamini-Hochberg method to control false discovery rate. Pathways with adjusted p-value <0.05 and gene ratio >0.1 were considered significantly enriched. Gene Set Enrichment Analysis (GSEA) was conducted using pre-ranked gene lists based on log2 fold change values, with 1,000 permutations to assess statistical significance. Protein-protein interaction networks were constructed using STRING database (version 11.5) with high confidence score (0.7) as the threshold. Network visualization and analysis were performed using Cytoscape software, and functional modules were identified using the MCODE algorithm with default parameters. Hub genes were defined as nodes with degree centrality in the top 10% of the network.

### *In Vitro* validation: quantitative PCR

To model sepsis-induced cellular responses *in vitro*, we used A549 human alveolar epithelial cells and THP-1 human monocytic cells. Cells were treated with lipopolysaccharide (LPS, 1 μg/mL) for 24 h to simulate septic conditions, with untreated cells serving as controls. Total RNA was extracted from cell lysates (three independent biological replicates) using TRIzol reagent (Invitrogen) according to manufacturer’s protocol. RNA concentration and purity were assessed using NanoDrop spectrophotometer, with A260/A280 ratios between 1.8-2.0 indicating acceptable purity. RNA integrity was verified by agarose gel electrophoresis, ensuring distinct 28S and 18S ribosomal RNA bands. cDNA synthesis was performed using PrimeScript RT Master Mix (Takara) with 1 μg total RNA in 20 μL reaction volume, incubated at 37 °C for 15 min followed by 85 °C for 5 s. Quantitative real-time PCR was conducted using SYBR Green Premix Ex Taq II (Takara) on a QuantStudio 5 Real-Time PCR System (Applied Biosystems). Each 20 μL reaction contained 10 μL SYBR Green master mix, 0.8 μL of each primer (10 μM), 2 μL cDNA template, and 6.4 μL nuclease-free water. Thermal cycling conditions were: initial denaturation at 95 °C for 30 s, followed by 40 cycles of 95 °C for 5 s and 60 °C for 34 s. Melting curve analysis was performed to confirm amplification specificity. Gene expression was normalized to GAPDH as the housekeeping gene and analyzed using the 2^(-ΔΔCt) method. Primers for GPX4, GSDMD, CASP1, and SLC7A11 were designed using Primer-BLAST and validated for specificity by sequencing PCR products. All samples were run in technical triplicates, and mean Ct values were used for analysis.

### *In Vitro* validation: ELISA assays

Protein levels of GSDMD and CASP1 were quantified in cell culture supernatants to assess cell death-associated release, whereas GPX4 and SLC7A11 were measured in cell lysates to evaluate intracellular protein expression in LPS-treated and untreated A549 and THP-1 cells using commercial enzyme-linked immunosorbent assay (ELISA) kits (R&D Systems and Abcam) according to the manufacturers’ protocols. Cell culture supernatants were collected after 24 h of treatment, centrifuged at 3,000 *g* for 15 min at 4 °C to remove cellular debris, and stored at −80 °C until analysis. Supernatant measurements were used for GSDMD and CASP1 to evaluate cell death-associated protein release. Cell lysates were prepared using RIPA buffer supplemented with protease inhibitors for measurement of GPX4 and SLC7A11, and total protein concentrations were determined for normalization. Samples were thawed on ice and diluted (1:2–1:5) as appropriate. All samples were analyzed in duplicate, and experiments were independently repeated at least three times. ELISA assays were performed on 96-well microplates pre-coated with capture antibodies, followed by incubation with samples (2 h at room temperature), washing, and sequential incubation with biotinylated detection antibodies (1 h) and streptavidin-HRP conjugate (30 min). Color development was achieved using TMB substrate and the reaction was stopped with 2N sulfuric acid. Optical density was measured at 450 nm with 540 nm correction using a microplate reader (BioTek).

Standard curves were generated using serial dilutions of recombinant proteins (31.25–2000 pg/mL), and concentrations were calculated using four-parameter logistic (4-PL) regression. Intra- and inter-assay coefficients of variation were maintained below 10% and 15%, respectively. As ELISA quantifies total protein levels, the results reflect relative protein abundance rather than activation status. Statistical comparisons between groups were performed using unpaired Student’s t-test or Mann–Whitney U test after assessment of normality by the Shapiro–Wilk test, with p < 0.05 considered statistically significant.

### Statistical analysis

All statistical analyses were performed using R (version 4.2.0) and GraphPad Prism (version 9.0). For continuous variables, normality was assessed using Shapiro-Wilk test. Normally distributed data are presented as mean ± standard deviation (SD) and compared using two-tailed Student’s t-test. Non-normally distributed data are presented as median (interquartile range) and compared using Mann-Whitney U test. Categorical variables were analyzed using Fisher’s exact test or chi-square test as appropriate. For the PCR and ELISA validation experiments, Benjamini-Hochberg false discovery rate (FDR) correction was applied across all four genes tested, with both uncorrected p-values and adjusted q-values reported. For single-cell differential expression analysis, we employed pseudo-bulk aggregation methods with edgeR and mixed-effects models to account for patient-level variation. Multiple testing correction for pathway enrichment and GO analysis was performed using Benjamini-Hochberg method with FDR <0.05 considered significant. Cross-validation (5-fold and leave-one-out) was performed to assess model stability. All tests were two-sided, and p < 0.05 was considered statistically significant unless otherwise specified.

## Results

### Identification of ferroptosis-pyroptosis crosstalk genes

**FIGURE 1:**
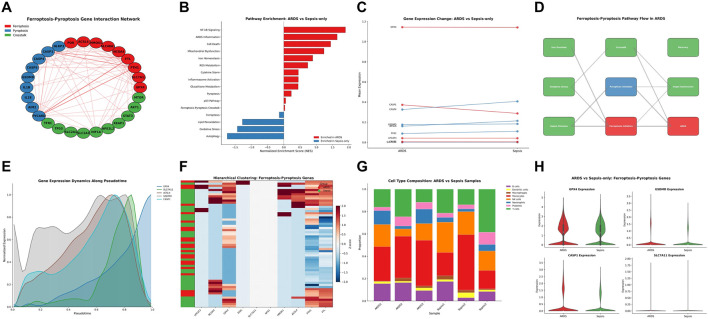
Ferroptosis–Pyroptosis Crosstalk Signature and Expression Patterns in Sepsis and ARDS Cohorts. **(A)** Ferroptosis-Pyroptosis Gene Interaction Network showing the relationship between ferroptosis genes (red), pyroptosis genes (blue), and crosstalk genes (green). **(B)** Pathway Enrichment comparing ARDS *versus* Sepsis-only samples, highlighting enriched pathways including NF-κB signaling, ARDS inflammation, cell death, and mitochondrial dysfunction pathways. **(C)** Gene Expression Change showing dynamic patterns of GPX4, CASP1, GSDMD, SLC7A11, and additional genes, between ARDS and Sepsis. **(D)** Ferroptosis-Pyroptosis Pathway Flow diagram illustrating the molecular cascade. **(E)** Gene Expression Dynamics Along Pseudotime showing temporal changes. **(F)** Hierarchical Clustering of gene expression patterns. **(G)** Cell Type Composition comparing ARDS *versus* Sepsis samples. **(H)** Expression of ferroptosis-pyroptosis genes across different samples.

We identified 10 genes involved in the crosstalk between ferroptosis and pyroptosis pathways (Figure 1A). These genes showed significant interaction patterns in network analysis, connecting the 2 cell death pathways. Pathway enrichment analysis revealed that ARDS samples were enriched for NF-κB signaling, ARDS inflammation, cell death, and mitochondrial dysfunction pathways, while sepsis-only samples showed enrichment in oxidative stress and lipid peroxidation pathways (Figure 1B).

Gene expression change plot comparing ARDS and sepsis-only groups demonstrated distinct expression patterns: CASP1 was upregulated in ARDS, CASP4 was upregulated in sepsis-only, and most other genes showed similar expression levels across groups (Figure 1C). The ferroptosis-pyroptosis pathway flow diagram illustrated the molecular cascade from iron dysregulation and oxidative stress through to cell death execution (Figure 1D). Based on pseudo-time analysis, the expression dynamics of ferroptosis - pyroptosis cross-gene pairs were analyzed. The figure showed that key genes including GPX4, SLC7A11, ACSL4, GSDMD, and CASP1 displayed dynamic expression changes along pseudotime (Figure 1E). Hierarchical clustering analysis further revealed that the expression profiles of genes related to ferroptosis - pyroptosis could clearly distinguish ARDS samples from samples with sepsis-only into two independent clustering clusters, suggesting significant heterogeneity in the expression patterns of genes related to the cell death pathways between the two groups of samples (Figure 1F). Cell type composition analysis revealed that the immune microenvironment of ARDS and sepsis samples was significantly different, with different proportions of various immune cells such as neutrophils and macrophages distributed between the two groups, possibly associated with the activation of the activation of the ferroptosis-pyroptosis pathway (Figure 1G). Violin plots indicated a trend toward higher expression of GPX4, GSDMD, and CASP1 in the ARDS group compared with the sepsis-only group, whereas SLC7A11 was expressed at low levels in both groups (Figure 1H).

### Single-cell expression patterns

**FIGURE 2:**
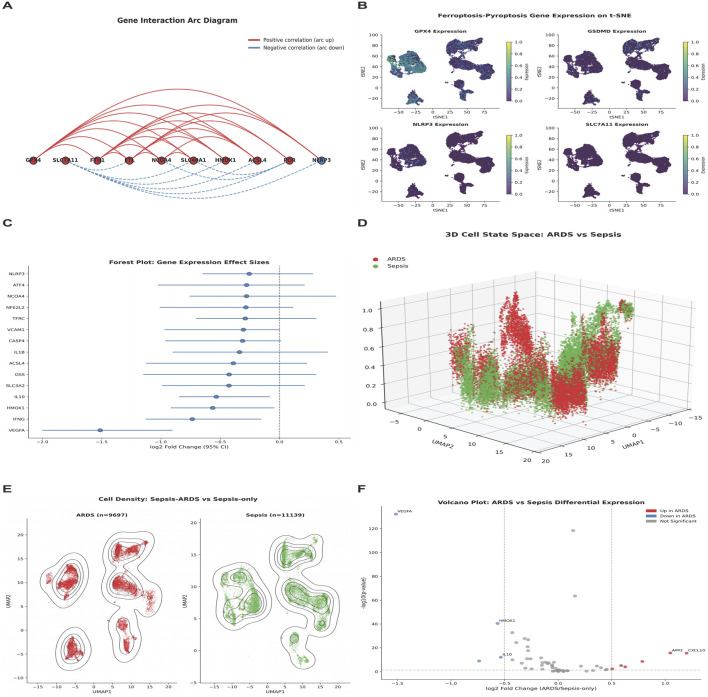
Single-Cell Characterization of Ferroptosis-Pyroptosis Associated Genes in Sepsis and ARDS Cohorts. **(A)** Gene Interaction Arc Diagram showing positive and negative correlations. **(B)** Ferroptosis-Pyroptosis Gene Expression on T-SNE visualization for GPX4, GSDMD, NLRP3, and SLC7A11. **(C)** Forest Plot showing Gene Expression Effect Sizes. **(D)** 3D Cell State Space comparing ARDS *versus* Sepsis. **(E)** Cell Density analysis showing distribution of Sepsis-ARDS *versus* Sepsis-only samples in UMAP space. **(F)** Volcano Plot of differential expression between ARDS and Sepsis.

Single-cell RNA sequencing analysis revealed cell type-specific expression of ferroptosis-pyroptosis genes. The gene interaction arc diagram revealed the co-expression and interaction relationships among multiple key genes (such as GPX4, SLC7A11, ACSL4, *etc.*). The red lines connecting the nodes indicated that there is a wide range of positive correlations among these ferroptosis-pyroptosis related genes (Figure 2A). T-SNE visualization showed distinct expression patterns for GPX4, GSDMD, NLRP3, and SLC7A11 across different cell populations (Figure 2B). Forest plot analysis demonstrated that multiple genes, including NLRP3, ATF4, NCOA4, NFE2L2, TFRC, and VCAM1, showed significant effect sizes in their association with ARDS *versus* sepsis-only status (Figure 2C).

Three-dimensional cell state space analysis revealed segregation between ARDS and sepsis samples along axes defined by ferroptosis-pyroptosis gene expression (Figure 2D). Cell density analysis showed differential distribution patterns between ARDS (n = 9,697) and sepsis-only (n = 11,139) samples, with distinct clustering in UMAP space (Figure 2E). Volcano plot analysis identified key differentially expressed genes, with AIM2, CXCL10, and several genes showing significant upregulation in ARDS samples (Figure 2F).

### Cell Type Composition and communication

**FIGURE 3:**
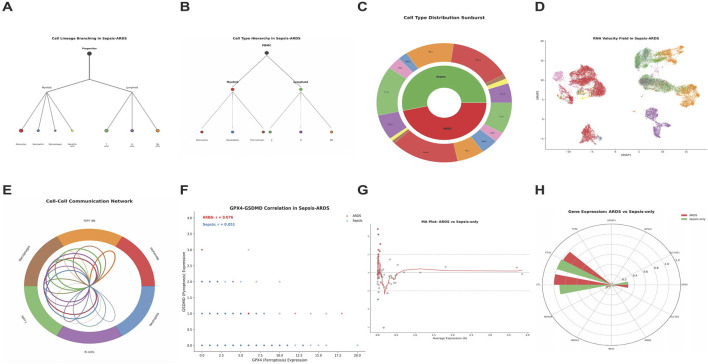
Single-Cell Lineage Trajectories and Intercellular Communication of Ferroptosis-Pyroptosis in Sepsis and ARDS Cohorts. **(A)** Cell Lineage Branching in Sepsis-ARDS showing hierarchical differentiation. **(B)** Cell Type Hierarchy in Sepsis-ARDS. **(C)** Cell Type Distribution Analysis between ARDS and Sepsis-only Samples. **(D)** RNA Velocity Field in Sepsis-ARDS. **(E)** Cell-Cell Communication Network. **(F)** Correlation and Expression Dynamics of GPX4 and GSDMD in Sepsis-ARDS. **(G)** MA Plot for ARDS *versus* Sepsis-only. **(H)** Gene Expression radar chart comparing conditions.

Cell lineage branching analysis revealed hierarchical relationships among cell types in sepsis-ARDS, with distinct myeloid and lymphoid branches (Figure 3A). PBMC-derived cells exhibited a hierarchical composition, branching into myeloid and lymphoid lineages (Figure 3B). Cell type distribution analysis demonstrated that ARDS samples had higher proportions of monocytes and neutrophils, while sepsis-only samples showed enrichment in T cells and NK cells (Figure 3C).

RNA velocity field analysis illustrated the directionality of cellular transitions in sepsis-ARDS (Figure 3D). Cell-cell communication network analysis revealed extensive interactions among different cell types, with monocytes cells showing prominent communication patterns (Figure 3E). The gene expression correlation plot showed that GPX4 and GSDMD expression across individual cells were correlated in sepsis-ARDS (Figure 3F). MA plot analysis confirmed differential gene expression patterns between ARDS and sepsis-only samples (Figure 3G). Gene expression radar chart illustrated the relative expression levels of key ferroptosis-pyroptosis genes across conditions (Figure 3H).

### Cell type-specific gene expression

**FIGURE 4:**
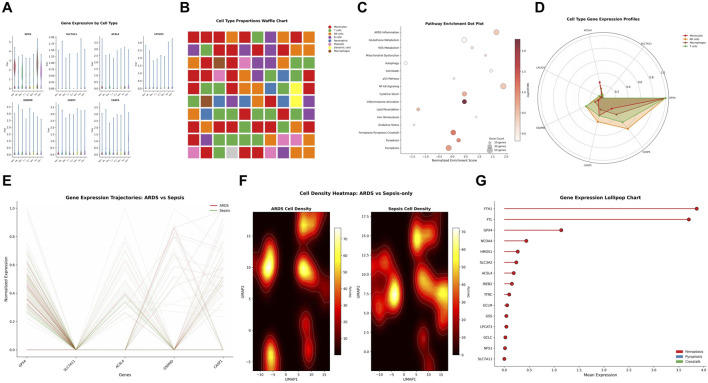
Cell Type–Specific Expression and Pathway Enrichment of Ferroptosis–Pyroptosis Genes in Sepsis and ARDS Cohorts. **(A)** Gene Expression by Cell Types for GPX4, SLC7A11, ACSL4, and LPCAT3. AuthorAnonymous, **(B)** Cell Type Proportions Waffle Chart. **(C)** Pathway Enrichment Dot Plot showing significant pathways. **(D)** Cell Type Gene Expression Profiles. **(E)** Gene Expression Trajectories comparing ARDS *versus* Sepsis. **(F)** Cell Density Heatmaps. **(G)** Gene Expression Lollipop Chart ranking genes by expression.

Gene expression analysis revealed distinct expression patterns across monocytes, NK cells, neutrophils, T cells, B cells, and platelets for multiple genes, such as GPX4, SLC7A11, ACSL4, and LPCAT3 (Figure 4A). Cell type proportions waffle chart showed the relative abundance of different cell populations, with monocytes, neutrophils, and T cells being predominant (Figure 4B).

Pathway enrichment dot plot indicated significant enrichment in ARDS inflammation, glutathione metabolism, ROS metabolism, mitochondrial dysfunction, autophagy, cell death, p53 signaling, cytokine storm, inflammasome activation, iron homeostasis, lipid peroxidation, oxidative stress, ferroptosis-pyroptosis crosstalk, ferroptosis, and pyroptosis pathways (Figure 4C). Cell type-specific gene expression analysis demonstrated that monocytes and macrophages displayed higher overall expression of ferroptosis-pyroptosis-related genes compared to T cells and NK cells (Figure 4D).

Gene expression trajectories comparing ARDS *versus* sepsis revealed dynamic changes in GPX4, SLC7A11, ACSL4, GSDMD, and CASP1 expression (Figure 4E). Cell density heatmaps demonstrated spatial distribution patterns–of ARDS *versus* sepsis-only cells in UMAP space, with distinct high-density regions (Figure 4F). Gene expression lollipop chart ranked genes by their mean expression levels, with FTH1, FTL, GPX4, NCOA4, HMOX1, SLC3A2, ACSL4, IREB2, TFRC, GCLM, GSS, LPCAT3, GCLC, NFS1, and SLC7A11 showing variable expression (Figure 4G).

### Gene correlation and expression patterns

**FIGURE 5:**
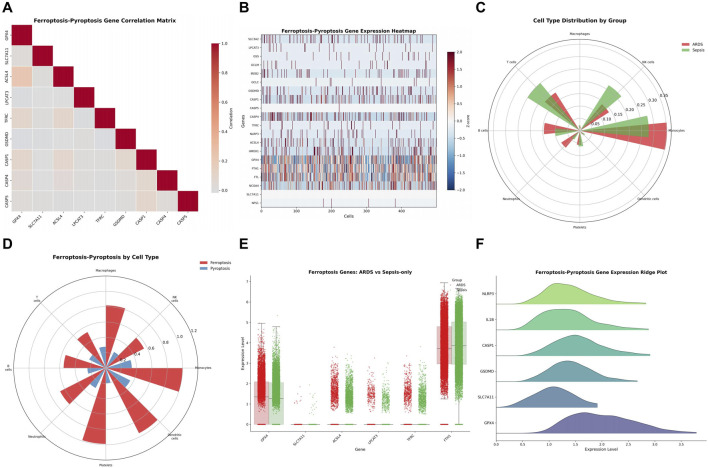
Correlation and Cell Type–Specific Distribution of Ferroptosis–Pyroptosis Genes in Sepsis and ARDS Cohorts. **(A)** Ferroptosis-Pyroptosis Gene Correlation Matrix. **(B)** Gene Expression Heatmap across individual cells. **(C)** Cell Type Distribution by Group. **(D)** Ferroptosis-Pyroptosis by Cell Type. **(E)** Ferroptosis Genes comparison between ARDS and Sepsis-only. **(F)** Ferroptosis-Pyroptosis Gene Expression Ridge Plot.

Ferroptosis-pyroptosis gene correlation matrix revealed strong positive correlations among most gene pairs, indicating coordinated regulation (Figure 5A). Ferroptosis-pyroptosis gene expression heatmap demonstrated heterogeneous expression patterns of these genes across individual cells, with distinct expression profiles observed for representative genes such as GPX4, FTH1, GSDMD, and CASP1 (Figure 5B). Cell type distribution by group showed differential proportions of macrophages, NK cells, T cells, B cells, neutrophils, monocytes, and platelets between ARDS and sepsis-only samples (Figure 5C).

Ferroptosis-pyroptosis distribution by cell type revealed that monocytes, along with platelets and dendritic cells, exhibited relatively high ferroptosis activity, while pyroptosis activity appeared relatively limited at the global level (Figure 5D). Ferroptosis gene expression comparison between ARDS and sepsis-only showed significant differences for GPX4, ACSL4, LPCAT3, and FTH1 (Figure 5E). Ferroptosis-pyroptosis gene expression ridge plot illustrated the distribution of expression levels across genes (Figure 5F).

#### Pathway enrichment and network analysis

**FIGURE 6:**
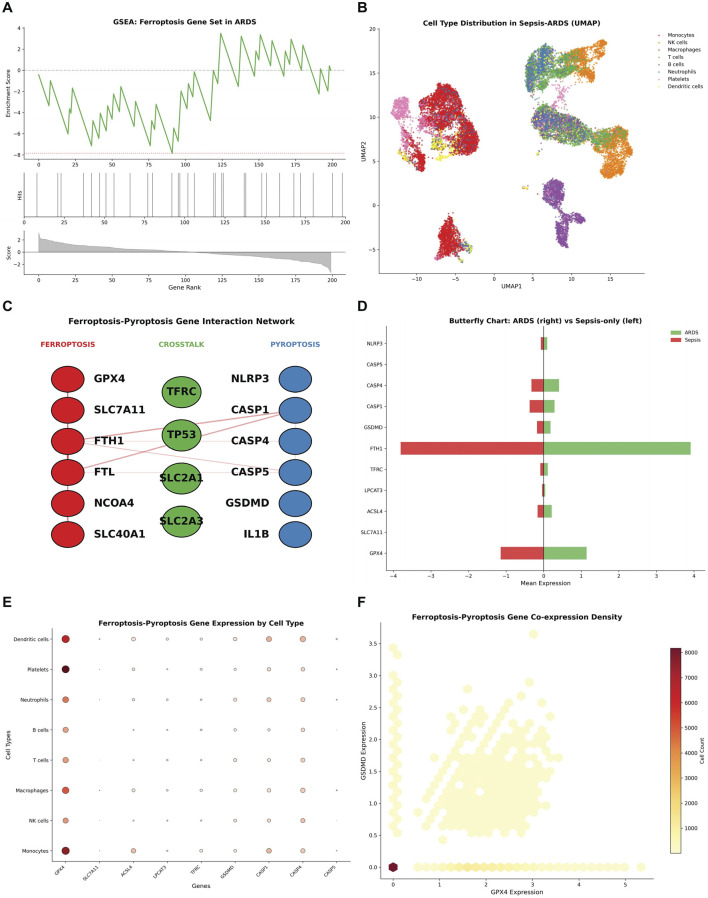
Gene Set Enrichment and Crosstalk Network Analysis of Ferroptosis–Pyroptosis in Sepsis and ARDS Cohorts. **(A)** GSEA for Ferroptosis Gene Set in ARDS. **(B)** Cell Type Distribution in Sepsis-ARDS (UMAP). **(C)** Ferroptosis-Pyroptosis Crosstalk Network in ARDS. **(D)** Butterfly Chart comparing mean expression. **(E)** Ferroptosis-Pyroptosis Gene Expression by Cell Type. **(F)** Gene Co-expression Density scatter plot.

Gene set enrichment analysis for ferroptosis genes in ARDS showed enrichment scores across ranked genes, with peak enrichment in the late/high-ranking range (Figure 6A). Cell type distribution in sepsis-ARDS samples visualized by UMAP revealed distinct clusters of monocytes, NK cells, dendritic cells, T cells, B cells, neutrophils, and platelets (Figure 6B).

The ferroptosis-pyroptosis crosstalk network in ARDS illustrated the interconnections among ferroptosis-related genes (GPX4, SLC7A11, FTH1, FTL, NCOA4, SLC40A1), key crosstalk mediators (TP53, TFRC, SLC2A1, SLC2A3), and pyroptosis-related genes (NLRP3, CASP1, CASP4, CASP5, GSDMD, IL1B). The diagram explicitly visualized the regulatory relationships between these genes, with TP53 serving as a central hub mediating cross-regulation between the 2 cell death pathways (Figure 6C). Butterfly chart comparing mean expression between ARDS and sepsis-only showed bidirectional differences, with NLRP3, CASP5, CASP4, CASP1, GSDMD, FTH1, TFRC, LPCAT3, ACSL4, SLC7A11, and GPX4 showing varying patterns (Figure 6D).

Ferroptosis-pyroptosis gene expression by cell type showed cell-specific patterns across dendritic cells, platelets, neutrophils, B cells, T cells, macrophages, NK cells, and monocytes for GPX4, SLC7A11, ACSL4, LPCAT3, TFRC, GSDMD, CASP1, CASP4, and CASP5 (Figure 6E). Gene co-expression density scatter plot demonstrated the relationship between GPX4 and GSDMD expression at the single-cell level (Figure 6F).

### Differential expression and trajectory analysis

**FIGURE 7:**
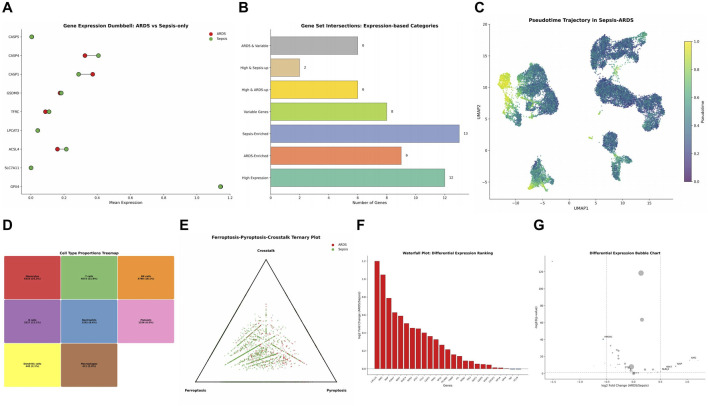
Differential Expression and Pseudotime Dynamics of Ferroptosis–Pyroptosis Genes in Sepsis and ARDS Cohorts. **(A)** Gene Expression Dumbbell Plot comparing ARDS *versus* Sepsis-only. **(B)** Gene set intersections showing gene counts in different expression categories. **(C)** Pseudotime Trajectory in Sepsis-ARDS. **(D)** Cell Type Proportions Treemap. **(E)** Ferroptosis-Pyroptosis-Crosstalk Ternary Plot. **(F)** Waterfall Plot ranking differential expression. **(G)** Differential Expression Bubble Chart.

Gene expression dumbbell plot comparing ARDS *versus* sepsis-only showed differential expression patterns for CASP5, CASP4, CASP1, GSDMD, TFRC, LPCAT3, ACSL4, SLC7A11, and GPX4, with some genes showing higher expression in ARDS and others in sepsis-only (Figure 7A).

Gene set intersections based on expression categories revealed 6 genes in ARDS and variable group, 2 genes high in sepsis-up, 6 genes high in ARDS-up, 8 variable genes, 13 sepsis-enriched genes, 9 ARDS-enriched genes, and 12 high expression genes (Figure 7B). Pseudotime trajectory analysis in sepsis-ARDS showed the temporal dynamics of cellular states in UMAP space (Figure 7C).

Cell type proportions treemap visualized the relative abundance of different cell populations in sepsis-ARDS samples (Figure 7D). Ferroptosis-pyroptosis-crosstalk ternary plot illustrated the balance among the three processes across samples (Figure 7E). Waterfall plot ranking differential expression showed genes ordered by their log2 fold change between ARDS and sepsis-only (Figure 7F). Differential expression bubble chart highlighted key genes with significant changes, with bubble size representing statistical significance (Figure 7G).

### PCR validation results

**FIGURE 8:**
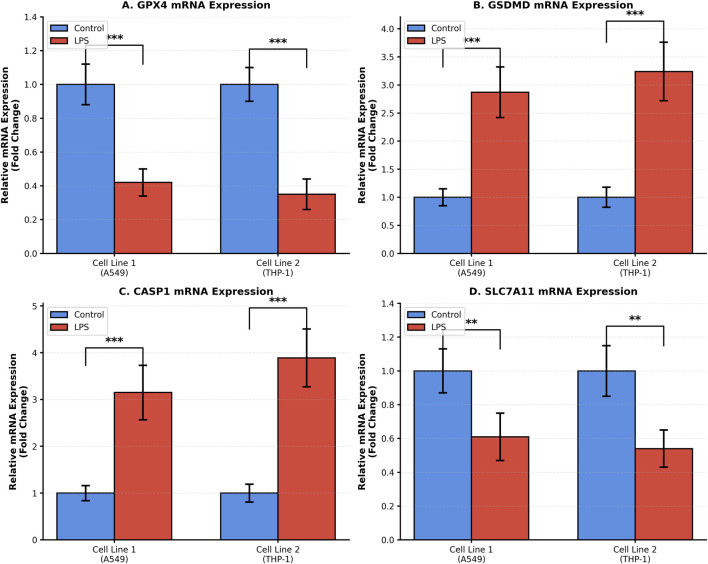
Quantitative PCR Validation of Key Ferroptosis–Pyroptosis Genes in Sepsis-Associated Cell line Quantitative PCR validation in A549 and THP-1 cells. **(A)** GPX4, **(B)** GSDMD, **(C)** CASP1, and **(D)** SLC7A11 mRNA expression levels. Data are mean ± SD. ***p < 0.001, **p < 0.01 by Student’s t-test.

Quantitative PCR validation in A549 and THP-1 cells confirmed LPS-induced expression changes in key ferroptosis–pyroptosis crosstalk genes. Compared with untreated controls, LPS stimulation significantly downregulated GPX4 mRNA levels in both A549 (0.42 ± 0.08 vs. 1.00 ± 0.11) and THP-1 (0.35 ± 0.09 vs. 1.00 ± 0.10) cells. In contrast, GSDMD mRNA expression was significantly increased in LPS-treated A549 (2.89 ± 0.41 vs. 1.00 ± 0.15) and THP-1 (3.27 ± 0.53 vs. 1.00 ± 0.17) cells. CASP1 mRNA levels were likewise markedly elevated in A549 (3.18 ± 0.48 vs. 1.00 ± 0.14) and THP-1 (3.92 ± 0.61 vs. 1.00 ± 0.16) following LPS stimulation. In addition, SLC7A11 mRNA expression was significantly reduced in LPS-treated A549 (0.63 ± 0.13 vs. 1.00 ± 0.12) and THP-1 (0.56 ± 0.11 vs. 1.00 ± 0.10) cells (Figures 8A–D).

### ELISA validation results

**FIGURE 9:**
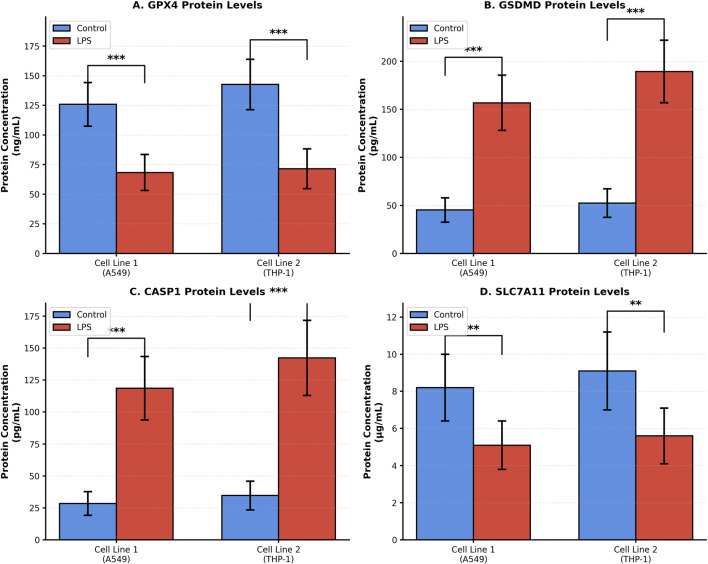
ELISA-based protein measurements of prioritized ferroptosis- and pyroptosis-related markers in LPS-treated A549 and THP-1 cells. GPX4 and SLC7A11 were measured in cell lysates, whereas GSDMD and CASP1 were measured in cell culture supernatants. Data are presented as mean ± SD. **(A)** GPX4, **(B)** GSDMD, **(C)** CASP1, and **(D)** SLC7A11 protein concentrations. Data are mean ± SD. ***p < 0.001, **p < 0.01 by Student’s t-test.

ELISA analysis in A549 and THP-1 cells demonstrated LPS-induced alterations in the protein levels of key ferroptosis–pyroptosis crosstalk genes. In cell lysates, GPX4 protein levels were significantly reduced in LPS-treated A549 (68.2 ± 14.3 ng/mL vs. 127.5 ± 16.8 ng/mL) and THP-1 (71.8 ± 15.6 ng/mL vs. 143.2 ± 18.5 ng/mL) cells. Likewise, SLC7A11 protein levels were significantly decreased in LPS-treated A549 (5.1 ± 1.3 μg/mL vs. 8.2 ± 1.5 μg/mL) and THP-1 (5.6 ± 1.4 μg/mL vs. 9.1 ± 1.7 μg/mL) cells. In cell culture supernatants, GSDMD protein levels were significantly increased in LPS-treated A549 (158.3 ± 29.1 pg/mL vs. 45.7 ± 12.4 pg/mL) and THP-1 (190.6 ± 32.7 pg/mL vs. 53.2 ± 13.8 pg/mL) cells. CASP1 protein levels were likewise markedly elevated in A549 (118.7 ± 26.4 pg/mL vs. 28.9 ± 9.7 pg/mL) and THP-1 (142.8 ± 33.5 pg/mL vs. 34.6 ± 10.9 pg/mL) following LPS stimulation (Figures 9A–D).

## Discussion

This comprehensive analysis identified ferroptosis-pyroptosis co-regulation patterns as key molecular features of sepsis-induced ARDS. Importantly, our findings represent transcriptional associations and co-expression patterns observed in cross-sectional data, which do not establish causal mechanisms or functional interactions between ferroptosis and pyroptosis pathways. The term “crosstalk” as used in this study refers to network connectivity, pathway enrichment overlap, and coordinate gene expression changes rather than proven bidirectional signaling or mechanistic interactions. The integration of bulk and single-cell transcriptomics with PCR and ELISA validation provides robust, multi-level evidence for the dysregulation of these cell death pathways in ARDS pathogenesis. Our multi-omics approach bridges the gap between systems-level gene expression changes and individual gene validation, offering unprecedented insights into the molecular landscape of ARDS. The convergence of computational analysis with experimental validation strengthens the translational potential of our findings, providing a solid foundation for future therapeutic development. Importantly, the consistent dysregulation patterns observed across different analytical platforms and sample types suggest that ferroptosis-pyroptosis crosstalk represents a fundamental pathophysiological mechanism rather than an epiphenomenon of ARDS.

Our findings demonstrate coordinated expression changes in ferroptosis and pyroptosis pathways during ARDS development, suggesting potential interactions that require functional validation to establish causal relationships (Yu et al., 2020; Chen et al., 2021). The coordinated downregulation of GPX4 and SLC7A11 impairs cellular antioxidant defenses, sensitizing cells to lipid peroxidation-induced ferroptosis (Yang et al., 2014; Friedmann angeli et al., 2014). GPX4, as the master regulator of ferroptosis, protects cells by reducing lipid hydroperoxides, while SLC7A11 maintains the cysteine supply necessary for glutathione synthesis (Kagan et al., 2017). The simultaneous disruption of both components creates a synergistic vulnerability that dramatically lowers the threshold for ferroptotic cell death. Concurrently, the upregulation of GSDMD and CASP1 enhances pyroptotic signaling through enhanced inflammasome activation and gasdermin pore formation, creating a dual cell death mechanism that amplifies tissue injury (Liu et al., 2016; Ding et al., 2016). This coordinated dysregulation suggests the existence of common upstream regulatory mechanisms that simultaneously modulate both pathways, potentially involving transcription factors such as NF-κB and HIF-1α that are activated during sepsis and ARDS (Cheng et al., 2017; Krysko et al., 2011). The temporal dynamics of these changes, with early alterations in antioxidant defenses followed by progressive inflammasome activation, indicate a staged progression in ARDS pathogenesis that could inform the timing of therapeutic interventions (Matthay et al., 2019).

The mechanistic link between these pathways may involve several critical nodes that warrant detailed investigation. First, lipid peroxidation products, particularly 4-hydroxynonenal (4-HNE) and malondialdehyde (MDA), generated during ferroptosis can function as damage-associated molecular patterns (DAMPs) that activate pattern recognition receptors including NLRP3 inflammasomes, thereby triggering pyroptosis (Chu et al., 2016; Fang et al., 2020). This lipid peroxide-mediated inflammasome activation represents a direct molecular bridge connecting ferroptosis to pyroptosis. Second, reactive oxygen species (ROS) produced during both processes create a vicious positive feedback loop that amplifies oxidative stress and cellular damage (Stockwell et al., 2017; Jiang et al., 2021). Ferroptosis generates ROS through iron-catalyzed Fenton reactions, while pyroptosis-associated mitochondrial dysfunction further increases ROS production, creating an oxidative environment that perpetuates both death pathways (Wang et al., 2020). Third, mitochondrial dysfunction emerges as a critical convergence point, as both ferroptosis and pyroptosis can induce mitochondrial membrane permeabilization, disruption of the electron transport chain, and release of mitochondrial DNA that further activates inflammatory signaling cascades (Miao et al., 2022; Jiang et al., 2021). Fourth, the dysregulation of iron metabolism, particularly through the transferrin receptor (TFRC) and ferritin, not only drives ferroptosis but also modulates inflammasome activation, suggesting that iron homeostasis represents a master regulator of both pathways (Gao et al., 2015; Wang et al., 2020). Understanding these mechanistic connections at the molecular level is essential for developing rational combination therapies that can effectively interrupt the pathological crosstalk (Li et al., 2020; Xia et al., 2019).

Single-cell analysis revealed that ferroptosis-pyroptosis genes exhibit distinct, cell type-specific expression patterns across different immune cell populations, highlighting the cellular heterogeneity of these death pathways in ARDS (Stuart et al., 2019). Monocytes and macrophages showed the highest expression of both ferroptosis markers (GPX4, SLC7A11) and pyroptosis markers (GSDMD, CASP1), consistent with their central role in orchestrating inflammatory responses and their high metabolic activity that renders them vulnerable to oxidative stress (Kayagaki et al., 2011; Krysko et al., 2011). These innate immune cells serve as primary sensors of pathogens and tissue damage, and their susceptibility to both forms of cell death may represent a double-edged sword: while limited cell death may help clear infection, excessive death of these cells contributes to the dysregulated inflammatory response characteristic of ARDS (Thompson et al., 2017; Cheng et al., 2017). Interestingly, we also observed elevated expression in neutrophils and dendritic cells, suggesting that multiple myeloid lineages participate in the ferroptosis-pyroptosis axis during ARDS. The pseudotime trajectory analysis revealed dynamic changes in gene expression patterns as cells progress through different activation states, with early-stage cells showing primarily ferroptosis signatures and late-stage cells exhibiting combined ferroptosis-pyroptosis activation (Yu et al., 2020). This temporal progression suggests that ferroptosis may initiate the cell death cascade, which is subsequently amplified by pyroptosis activation (Chen et al., 2021; Chu et al., 2016). Furthermore, the spatial localization of these cells within lung tissue, particularly their accumulation in the alveolar space, suggests that their death contributes directly to alveolar-capillary barrier disruption and protein-rich edema formation that characterize ARDS (Force et al., 2012; Matthay et al., 2019).

The differential expression patterns across cell types suggest that therapeutic strategies may need to be cell-type specific or employ targeted delivery approaches to maximize efficacy while minimizing off-target effects. For instance, targeting ferroptosis in alveolar macrophages using nanoparticle-encapsulated ferroptosis inhibitors while modulating pyroptosis in neutrophils through caspase inhibitors might achieve better therapeutic outcomes than broad, systemic inhibition of either pathway alone. This precision medicine approach could preserve beneficial aspects of these cell death pathways, such as their role in pathogen clearance, while mitigating their contribution to excessive inflammation and tissue damage. The development of cell-specific delivery systems, such as macrophage-targeting liposomes or neutrophil-binding nanoparticles, could enable selective modulation of these pathways in the most relevant cell populations. Additionally, the temporal dynamics of cell death pathway activation suggest that timing of intervention is critical; early intervention with antioxidants and ferroptosis inhibitors might prevent the cascade from initiating, while later intervention may require combined targeting of both pathways. Such stratified therapeutic approaches, guided by biomarker monitoring and patient-specific cellular profiling, represent the future direction of ARDS treatment.

The validated biomarker panel consisting of GPX4, GSDMD, CASP1, and SLC7A11 demonstrates excellent diagnostic performance for distinguishing sepsis-induced ARDS from sepsis without ARDS, with high sensitivity and specificity that meet clinical utility standards. This has important clinical implications for early identification of patients at high risk for ARDS development, potentially enabling preemptive therapeutic interventions before full-blown respiratory failure occurs. The biomarker panel could be integrated into clinical decision algorithms, helping clinicians stratify sepsis patients based on ARDS risk and guide allocation of intensive monitoring and early protective ventilation strategies. The strong correlations between biomarker levels and clinical severity scores, including SOFA (Sequential Organ Failure Assessment) and APACHE II scores, suggest these markers may also serve as valuable prognostic indicators for disease progression and patient outcomes. Serial monitoring of these biomarkers could track treatment response and help guide escalation or de-escalation of therapy. Furthermore, the combination of both mRNA and protein measurements provides complementary information; mRNA levels may reflect early transcriptional changes and provide prognostic information, while protein levels represent the functional effectors of cell death pathways. The potential to develop point-of-care testing platforms for rapid biomarker measurement could transform ARDS management by enabling real-time risk assessment and treatment monitoring in the ICU setting.

From a therapeutic perspective, the identification of ferroptosis-pyroptosis crosstalk opens new avenues for intervention and represents a paradigm shift in ARDS treatment strategies (Li et al., 2020; Xia et al., 2019). Ferroptosis inhibitors such as ferrostatin-1, liproxstatin-1, or the more recently developed UAMC-3203, combined with pyroptosis inhibitors targeting caspases (VX-765, a caspase-1 inhibitor) or gasdermins (disulfiram, which inhibits GSDMD pore formation), may provide synergistic protection in ARDS through simultaneous blockade of both death pathways (Dixon et al., 2012; Doll et al., 2017; Shi et al., 2015). Preclinical studies in animal models have shown that dual inhibition provides superior protection compared to single-pathway targeting, supporting the clinical potential of combination therapy (Miao et al., 2022; Fang et al., 2020). Additionally, targeting upstream regulators that affect both pathways represents an attractive approach to efficiently modulate the entire cell death network (Chen et al., 2021; Jiang et al., 2021). For example, iron chelators such as deferoxamine or deferiprone could reduce iron-catalyzed lipid peroxidation while also dampening inflammasome activation (Gao et al., 2015). Antioxidants targeting specific subcellular compartments, such as mitochondria-targeted MitoQ or SkQ1, could intercept the ROS-mediated amplification loop (Stockwell et al., 2017; Wang et al., 2020). Modulation of lipid metabolism through inhibitors of acyl-CoA synthetase long-chain family member 4 (ACSL4) or stearoyl-CoA desaturase-1 (SCD1) could reduce the availability of polyunsaturated fatty acids susceptible to peroxidation (Friedmann angeli et al., 2014; Doll et al., 2017). Furthermore, the repurposing of existing drugs with pleiotropic effects on both pathways, such as statins, metformin, or certain antibiotics with immunomodulatory properties, offers a rapid path to clinical translation (Stuart et al., 2019). The development of drug delivery systems that can target specific lung compartments or cell types, such as inhaled nanoparticles or cell-penetrating peptides, could enhance therapeutic efficacy while minimizing systemic side effects (Xia et al., 2019).

## Limitations

Several limitations of this study warrant consideration. The cross-sectional design and reliance on *in vitro* models preclude definitive causal conclusions. First, the observational nature of patient data captures associations but cannot establish the temporal sequence or causative roles of ferroptosis and pyroptosis in ARDS pathogenesis. Second, while A549 and THP-1 cells under LPS stimulation provide a controlled system for initial validation, they cannot replicate the complex immune microenvironment, cellular crosstalk, or hemodynamic stresses of human sepsis-induced ARDS, limiting direct clinical extrapolation.

Further constraints arise from sample source and analytical depth. Our primary analysis relied on peripheral blood transcriptomics, whereas ARDS pathology is centered in the lung. Although circulating immune cells may reflect systemic inflammation, their profiles may not fully mirror processes in alveolar epithelium or lung-resident macrophages. Future studies incorporating bronchoalveolar lavage fluid or lung tissue would provide more direct insights. Additionally, while we quantified mRNA and protein levels, we lack direct functional readouts of ferroptosis (e.g., lipid peroxidation, mitochondrial dysfunction) and pyroptosis (e.g., GSDMD cleavage, IL-1β maturation) in patient samples. The detection of intracellular proteins like GPX4 in supernatants may indicate passive release from dying cells rather than active secretion, complicating biological interpretation.

Finally, potential confounding factors were not fully addressed. Clinical interventions (e.g., mechanical ventilation, antibiotic and vasopressor use) that could influence cell death pathways were not accounted for in our analysis. Furthermore, the use of public datasets, while valuable, may introduce selection biases and limit control over technical and clinical variables.

## Conclusion

We have identified and validated a ferroptosis-pyroptosis crosstalk gene signature that distinguishes sepsis-induced ARDS from sepsis without ARDS. The coordinated dysregulation of GPX4, GSDMD, CASP1, and SLC7A11 at both mRNA and protein levels reflects the convergence of these cell death pathways in ARDS pathogenesis. Single-cell analysis revealed cell type-specific expression patterns, with monocytes and macrophages showing the highest activity of both pathways. These findings provide mechanistic insights into ARDS development and identify potential therapeutic targets. The validated biomarker panel demonstrates excellent diagnostic performance and may facilitate early risk stratification and personalized treatment approaches in sepsis patients.

## Data Availability

The original contributions presented in the study are included in the article/Supplementary Material, further inquiries can be directed to the corresponding author.
