# Supplementary material for: Ferroptosis-pyroptosis crosstalk signature as prognostic biomarkers and therapeutic targets in sepsis-induced ARDS
Source: Front Cell Dev Biol. 2026 May 15;14:1810674. doi: 10.3389/fcell.2026.1810674 (PMC13219286; doi:10.3389/fcell.2026.1810674)
Supplement: Supplementary file 1 [file Table1.docx]

**Supplementary Table S1: Donor-Level Cell Contributions in Single-Cell RNA Sequencing Analysis**

**Dataset: GSE151263 (Peripheral Blood Mononuclear Cells)**

| **Patient ID** | **Condition** | **Age** | **Sex** | **Cells After QC** | **Detected Genes** | **Mito %** | **SOFA** |
| --- | --- | --- | --- | --- | --- | --- | --- |
| SEP001 | Sepsis | 62 | M | 2,847 | 1,432 | 8.2% | 8 |
| SEP002 | Sepsis | 54 | F | 3,156 | 1,389 | 7.9% | 7 |
| SEP003 | Sepsis | 71 | M | 2,684 | 1,512 | 9.1% | 9 |
| ARDS001 | ARDS | 58 | M | 3,421 | 1,654 | 8.7% | 12 |
| ARDS002 | ARDS | 65 | F | 3,189 | 1,598 | 9.3% | 14 |
| ARDS003 | ARDS | 49 | M | 3,087 | 1,623 | 8.5% | 13 |

**Total Cells:**

• Sepsis-only: 8,687 cells (3 patients)

• ARDS: 9,697 cells (3 patients)

**Quality Control Metrics:**

• Genes detected per cell: 200-6000 (range)

• Mitochondrial gene percentage: <15%

• Doublet removal: DoubletFinder applied

• Batch correction: Harmony algorithm

**Sensitivity Analysis:**

Downsampled datasets (equalizing to 8,687 cells per group) showed consistent differential expression patterns with the full dataset (Spearman correlation = 0.94, p < 0.001).

**Notes:**

• All patients met Sepsis-3 criteria

• ARDS patients met Berlin criteria (moderate to severe)

• Primary infection sources: pneumonia (n=3), abdominal sepsis (n=2), urinary tract infection (n=1)

• Samples collected within 48 hours of sepsis/ARDS diagnosis

• No patients received ferroptosis or pyroptosis-modulating therapies prior to sampling
