# Supplementary material for: Ferroptosis-pyroptosis crosstalk signature as prognostic biomarkers and therapeutic targets in sepsis-induced ARDS
Source: Front Cell Dev Biol. 2026 May 15;14:1810674. doi: 10.3389/fcell.2026.1810674 (PMC13219286; doi:10.3389/fcell.2026.1810674)
Supplement: Supplementary file 2 [file Table2.docx]

**Supplementary Table S2: Quantitative Rankings for Ferroptosis-Pyroptosis Crosstalk Gene Prioritization**

All 10 identified crosstalk genes with metrics used for selecting the 4-gene validation panel (GPX4, GSDMD, CASP1, SLC7A11)

| **Gene** | **Betweenness** | **Degree** | **Log2FC** | **Detection** | **Selected** | **Rationale** |
| --- | --- | --- | --- | --- | --- | --- |
| GPX4 | 0.245 | 18 | 2.13 | 45.2% | **YES** | All criteria met |
| GSDMD | 0.198 | 16 | 1.87 | 42.8% | **YES** | All criteria met |
| CASP1 | 0.224 | 15 | 1.92 | 38.5% | **YES** | All criteria met |
| SLC7A11 | 0.187 | 14 | 1.78 | 36.9% | **YES** | All criteria met |
| ACSL4 | 0.156 | 12 | 1.42 | 32.1% | NO | Failed log2FC criterion |
| NLRP3 | 0.143 | 11 | 1.38 | 28.7% | NO | Failed log2FC and detection |
| LPCAT3 | 0.128 | 10 | 1.29 | 26.4% | NO | Failed log2FC and detection |
| PTGS2 | 0.112 | 9 | 1.15 | 24.8% | NO | Failed multiple criteria |
| ALOX15 | 0.095 | 8 | 1.08 | 22.3% | NO | Failed multiple criteria |
| NOX1 | 0.081 | 7 | 0.94 | 19.5% | NO | Failed multiple criteria |

**Selection Criteria (Pre-specified):**

1. Betweenness Centrality ≥0.15 (90th percentile threshold)

2. Degree Centrality ≥12 (top quartile threshold)

3. |log2 Fold Change| >1.5 in bulk RNA-seq (GSE65682)

4. Detection Rate ≥30% in single-cell RNA-seq (GSE151263)

**Network Construction Parameters:**

• STRING database version 11.5

• Confidence score threshold: 0.7 (high confidence)

• Network analysis: Cytoscape 3.9 with NetworkAnalyzer plugin

• Total nodes in ferroptosis-pyroptosis network: 538 genes

• Total edges: 2,847 interactions

**Notes:**

• Rankings are based on integrated multi-omics data (network topology + expression changes + single-cell detection)

• The 4-gene panel was selected using pre-specified quantitative criteria before experimental validation

• All criteria were defined prior to PCR and ELISA experiments to avoid selection bias

• Future validation of the remaining 6 genes is planned for an independent cohort study
